# Special Issue “Rain Sensors”

**DOI:** 10.3390/s23156934

**Published:** 2023-08-04

**Authors:** Filippo Giannetti, Luca Giovanni Lanza

**Affiliations:** 1 Department of Information Engineering (DII), University of Pisa, 56122 Pisa, Italy; 2 Department of Civil, Chemical and Environmental Engineering (DICCA), University of Genova, 16145 Genoa, Italy; luca.lanza@unige.it; 3 WMO Measurement Lead Centre “B. Castelli” on Precipitation Intensity, 16145 Genoa, Italy

## 1. Introduction

In situ weather sensors aiming at the measurement of liquid atmospheric precipitation (rainfall) experienced limited conceptual innovation in recent decades, except for the data recording and transmission components. The most relevant advancement in modern rainfall measurement devices remains the transition from catching to non-catching instruments. The former are used to measure the integral properties of rainfall (the total amount and intensity of the rainwater collected through a funnel), whereas recent sensors (most of them contactless) can detect the microphysical features of the rainfall process (e.g., the drop size distribution and fall velocity) or the kinetic energy of each drop. These sensors are based on modern measurement techniques and involve optical, acoustic and radar measuring principles, rather than the more traditional gravimetric (see, e.g., Cauteruccio et al. [1]) and seldom adopted thermodynamic ones (see, e.g., Cauteruccio et al. [2]).

However, the lack of any standardized calibration procedure for non-catching instruments (Lanza et al. [3]) and the differences still observed between measurements from co-located modern and traditional (reference) instruments (see, e.g., Lanza and Vuerich [4]) still make the reliability and accuracy of the new sensors largely questionable. The role of external influencing variables, such as the environmental conditions at the measurement site, is also rarely accounted for, and no adjustments are usually applied to the raw data to correct, e.g., for the wind-induced bias (Chinchella [5]). Therefore, their use in applications out of the research framework should not be encouraged before sufficient knowledge of both the instrumental and environmental sources of bias is achieved and suitable adjustments are implemented.

The development of opportunistic sensors is the second main innovation recently experienced, suggesting a high potential for large-scale application due to the low cost of their installation and operation. These sensors exploit already-existing, but usually unrelated, microwave (MW) or millimeter wave (mmW) links to infer the rainfall amount or intensity by interpreting the extra attenuation induced by the precipitation on the received signal level. Communication technologies that can be opportunistically used for rain sensing include commercial MW links (CMLs) of cellular phone networks, satellite MW links (SMLs), including broadcast satellite links (BSLs), but also wireless sensor networks (WSNs), e.g., for Internet of things (IoT) applications, moving vehicles, surveillance cameras, etc. (see, e.g., Uijlenhoet et al. [6], Giannetti et al. [7], Haberlandt and Sester [8] and Allamano et al. [9]). These sensors turn out appropriate for large-scale installation (e.g., within citizen scientist initiatives) and coverage of wide areas, where in situ rainfall measurements are rarely sufficient or even possible. However, the availability of comprehensive and convincing validation exercises is still scarce (see, e.g., Colli et al. [10]) and assessing their accuracy is difficult. Indeed, opportunistic sensors in most cases provide cumulative information about rainfall along some geometric path (from linear to unpredictable), which does not match the coverage of any traditional instrument that could be used as a reference.

Weather radars and remote sensors on board meteorological satellite platforms have been largely developed and used to measure rainfall over wide areas, with increasingly enhanced spatial and temporal resolution. These are based on various remote imaging systems operating in different bands of the light spectrum (from visible to MW wavelengths). Their main advantage is the spatial coverage of wide geographical areas, with the capability to provide areal averaged estimates of the rainfall field over hydrological units (the catchment area) where many applications operate (e.g., flood forecasting, water resources management, etc.). However, the associated rainfall products largely rely on in situ rainfall measurements for calibration and validation purposes. Following Michaelides et al. [11] “measurements at the ground have been proved indispensable, despite advances in several areas of remotely sensing of precipitation”.

To provide specific guidance about instrument calibration and their achievable accuracy, perform laboratory and field tests, and develop research/technical activities about the measurement of precipitation intensity and the related data analysis and interpretation, the first Measurement Lead Centre on Precipitation Intensity was designated by the World Meteorological Organization (WMO) in Italy in 2010 [12]. The Lead Centre is dedicated to the memory of, and named after, Benedetto Castelli and his historical work on precipitation measurements. A reference system for rainfall intensity observations is available at the field test site of the Lead Centre in Vigna di Valle (Rome, Italy) for testing the performance of various existing and new rain sensors. Installed in a pit, it is composed of a set of working reference instruments employing a selection of measuring principles tested during the previous WMO Field Intercomparisons of Rainfall Intensity Gauges (see Lanza and Vuerich [4]).

## 2. Overview of Contributions

This Special Issue (SI) collects papers about rain sensor technologies and applications, with the goal of providing the readership with an understanding of operating principles, state-of-the-art, applications, and future trends of such devices. Contributions were provided by researchers from both academia and industry on many different aspects of rain sensor technologies and applications, spanning over different measuring principles, measurement scales, and the measured characteristics of the rainfall process (intensity, drop size distribution, fall velocity, etc.). Papers touch upon on the various aspects of sensor calibration, uncertainty assessment, standardization, and validation.

This SI includes seventeen contributions: two of them are in the form of surveys, while the remaining fifteen are feature papers presenting the state-of-the-art of the technology, applications, case studies, prototypes, results of measurement campaigns, and innovative solutions for rain sensing. The devices and technologies addressed are:

- Catching rain gauges;
- Non-catching rain gauges (disdrometers; laser precipitation monitors; piezoelectric sensors);
- Weather radars and spaceborne remote sensing platforms;
- Opportunistic rain sensing (CML, SML/BSL, IoT);
- Gamma-dose rate monitoring.

Author affiliations are from nineteen countries across all continents (Africa, North and South America, Asia, Europe and Oceania) and the papers present experimental results obtained in seven countries (China, Italy, Kenya, Mexico, Poland, Puerto Rico–USA, and Spain).

## 3. Contributions

### 3.1. Rain Gauges

An empirical assessment of the impact of wind on accumulated precipitation measurements in Poland is presented by Barańczuk et al. [13], who analyzed daily precipitation data obtained from two catching type instruments, the Hellman totalizer rain gauge and the GGI 3000 evaporimeter set (with collector areas varying from 200 to 3000 cm^2^, respectively). The cylindrical, Hellman gauge is positioned on a pole at 1.0 m above the ground while the evaporimeter, still cylindrical in shape, is positioned at 0.05 m above the ground. Though the height range is limited, a significant difference of 3–4% was observed in the collected precipitation at the yearly scale, which the authors attribute entirely to the aerodynamic impact of wind on the sensor.

Stagnaro et al. [14] performed dynamic calibration in the laboratory on two catching type drop counter instruments and applied the developed adjustment curve to real-world measurements from a field test site at the Hong Kong Observatory. The rainfall climatology at the site was found to be crucial in determining the magnitude of the measurement bias, with a predominant overestimation at the low to intermediate rainfall intensity range. This work demonstrates the relevance of the instrumental sources of bias even for sensors adopting quite simple technologies (optical in this case), and the need for accurate calibration before operational use.

The accuracy and calibration issues of non-catching instruments for in situ rainfall measurements are addressed in a couple of papers. Baire et al. [15] focus on quantifying the instrumental bias and present a traceable method for their calibration, using high precision rain drop generators. Three different European institutes, National Standards (Belgium), Danish Technological institute (Denmark) and the University of Genova (Italy) developed their own version of the calibration device and presented an estimate of the associated calibration uncertainty. The work is propaedeutic to the development of standardized procedures, which are presently under discussion within the European Standardization Body (CEN), Technical Committee 318 (Hydrometry).

On the other hand, Chinchella et al. [16] investigated the environmental bias due to the influence of wind on rainfall measurements obtained from the Thies LPM instrument, a laser disdrometer providing measurements of both raindrop size and fall velocity. Results suggest a strong impact of wind on the measurement accuracy, depending on the wind speed and direction, since the gauge body is not radially symmetric and cannot be easily aligned with the prevailing wind at the site.

In the paper by Antonini et al. [17], the development of a prototype of an impact rain gauge based on a very low-cost piezoelectric sensor was presented. Machine learning methods were tested and compared for the calibration of the relationship between the different properties of the voltage signal, as sampled by the rain drop impact, and rainfall intensity. Although some potential is evident, additional validation is still needed, especially in different seasons and on different types of precipitation.

### 3.2. Weather Radars and Spaceborne Platforms

A statistical cross-evaluation study of the radar reflectivity from the dual-frequency precipitation radar (DPR) onboard the Global Precipitation Measurement Mission (GPM) and the U.S. National Weather Service (NWS) Next-Generation Radar (NEXRAD) ground-based instrument was performed by Acosta-Coll et al. [18] over the island of Puerto Rico (USA). The GPM at Ku-band and Ka-band and NEXRAD at S-band overlapping scanning regions were compared during typical weather precipitation events and during four extreme weather events. The authors found that the correlation coefficient between the two instruments during the analyzed extreme weather events (including tropical cyclones) was moderate to low, depending on the elevation angle of the ground-based radar.

A method for rain area detection and correction from reflectance and infrared (IR) brightness temperatures data of the Meteosat Second Generation (MSG) satellite are presented by Kingsley et al. [19]. A gradient based adaptive correction technique was developed to reduce the number and size of the detected rain areas. After calibration and validation with rainfall data from a dense network of ground-based rain gauge stations in southwestern Kenya, the model showed good agreement in the spatial dynamics of the detected rain area and rain rate.

### 3.3. Opportunistic Sensing Techniques

Due to rainfall’s high spatial and temporal dynamics, accurate real-time monitoring of rain intensity and retrieval of the rain map are very challenging tasks. To this respect, rain gauges, weather radars, and satellite remote sensing, i.e., the three main rainfall measurement methods at present, exhibit some limitations and drawbacks. In the last few decades, the use of pre-existing MW (or mmW) communication links (i.e., dedicated to other communication services), as sensing signals gained increasing attention and emerged as a very promising technique capable of providing rainfall estimates with high spatial and temporal resolution. The basic idea for this approach to rain sensing, termed opportunistic, relies on: (i) measuring the extra attenuation of the signal caused by the presence of precipitation along some MW (or mmW) propagation path, and (ii) mapping this attenuation into a rain rate value, via signal processing based on some tropospheric model, e.g., the popular power law relationship between the rainfall rate (in mm/h) and the rain-induced attenuation (in dB).

The paper by Lian et al. [20] contains a comprehensive survey on rainfall estimation based on opportunistic measurement of the extra attenuation introduced by the rain on CMLs, such as the fronthaul/backhaul communications among base stations of mobile networks. The paper introduces the basic principle of this technique, reviewing the state-of-the-art solutions for the different steps of signal processing, also addressing uncertainties and errors involved in each step, as well as their impacts on the accuracy of rainfall measurement. Challenges and future directions are eventually discussed.

Nebuloni et al. [21] assess the performance of a network of CMLs as opportunistic rainfall sensors in a challenging mountainous environment in Northern Italy. The benchmark dataset was provided by a set of rain gauges and disdrometers. Data gathered from disdrometers were first used to calibrate the power law relationship, then CML-based rainfall estimates were derived and compared with rainfall sensor measurements. Experimental results show that CML opportunistic technique is effective in rainfall detection, whereas its quantitative rainfall estimates may exhibit large discrepancies.

Song et al. [22] used eight MW links (operating at 15 GHz and 23 GHz) for real-time rain rate estimation in Jiangyin (Eastern China). A strong positive between the rain-induced attenuation of MW links and the rain rate measured by rain gauges was demonstrated. Furthermore, real-time results indicate that MW links estimate the rainfall with a higher temporal resolution than customary rain gauges.

The paper by Zheng et al. [23] introduces a field experiment using a 3 km long link in the E-band (71–81 GHz) of the mmW range to obtain rainfall rate information in Nanjing city (Eastern China). The implemented algorithm first distinguishes between the wet and the dry periods, then determines the classification threshold for calculating attenuation baseline in real time, corrects the influence of the wet antenna attenuation and finally calculates the rainfall rate through the power law relationship between the rainfall rate and the rain-induced attenuation. Experimental results, expressed in terms of correlation and relative error between the rainfall rate retrieved from the mmW link and that measured by a raindrop spectrometer, demonstrate the reliability and accuracy of the proposed rainfall monitoring technique.

A different approach to opportunistic rain sensing is based on the measurement of the attenuation induced by rain on SMLs, either BSLs for direct-to-home TV reception from geostationary (GEO) satellites in Ku (10–13 GHz) and Ka (17–20 GHz) bands, or signals from low-Earth orbit (LEO) satellites belonging to large constellations for global broadband access.

Giannetti and Reggiannini [24] present a broad overview of the numerous issues inherent in the SML-based rain monitoring approach, along with a number of solutions and algorithms proposed in the literature in recent years, and ultimately provide an exhaustive account of the current state of the art on this topic.

Pastoriza-Santos et al. [25] experimentally outline some of the characteristics and drawbacks affecting the propagation of a radio beacon in Ka-band from a GEO satellite to a ground-based measurement equipment, made of low-cost commercial-grade devices, as in case of SML-based opportunistic rain sensing. The paper describes a procedure for evaluating and subtracting the baseline level corresponding to no-rain conditions from the raw received beacon signal. The impact of some meteorological parameters and the unavoidable (though small) antenna pointing errors are presented, too.

In the paper by Shen et al. [26], dual-polarized MW signals from LEO satellites are employed for raindrop size distribution retrieval. The feasibility of this approach is studied by modelling and simulating the retrieval system, including multiple ground receivers equipped with signal-to-noise ratio estimators and a LEO satellite communicating with the receivers using both vertically and horizontally polarized signals. Simulation results confirm that the specific attenuation ratio of vertically to horizontally polarized signals can be effectively used to retrieve the slope and intercept parameters of raindrop size distribution.

Saggese et al. [27] propose a novel algorithm to estimate the rainfall rate map from attenuation measurements on both BSLs and CMLs. The approach pursued therein extends the well-known two-dimensional GMZ (Goldshtein–Messer–Zinevich) algorithm to fuse the attenuation data coming from different links in a three-dimensional scenario. Simulation results prove the convergence of the procedures and show that adding the BSL links to a pre-existent CML network boosts the accuracy performance of the estimated rainfall map.

Another opportunistic technique for rain sensing consists of the measurement of the attenuation induced by rain on WSNs. To this respect, Gutiérrez-Gómez et al. [28] developed a novel sounding system based on the measurement of point-to-point long range (LoRa) chirp radio signals in the 915 MHz industrial, scientific and medical (ISM) band. The system was tested in a case study close to and over water in a tropical meadow region, the Colima River in Mexico. The results show that the LoRa signal propagation over water exhibits a log-normal distribution. Furthermore, two new experimental path loss models are presented.

### 3.4. Non-Conventional Techniques

Yakovleva et al. [29] used measurements of 𝛾-radiation dose rate ambient equivalent performed with a scintillation detector BDKG-03 (at 1 min sampling rate) to confirm that a change in the intensity of precipitation leads to a change in the 𝛾-radiation dose rate increase speed (time derivative). The authors propose a method of estimating the average value of the intensity and amount of precipitation for one event, reconstructing the intensity spectrum from experimental data on the dynamics of the measured dose rate of 𝛾-radiation. Reconstruction of various events and comparison with ground-based measurements using a Davis Rain Collector II and an optical (laser) precipitation gauge, named OPTIOS, show the good performance of the proposed method.
